# Rare Structural Variants Uncovered by Optical Genome Mapping in Multisystem Inflammatory Syndrome in Children (MIS‐C)

**DOI:** 10.1002/ggn2.202500023

**Published:** 2025-12-08

**Authors:** Catherine A. Brownstein, Caspar I. van der Made, Kristin Cabral, Shira Rockowitz, Donghun Kang, Maximilian Schieck, Andy Wing Chun Pang, Jeffrey M. Robinson, Alex R. Hastie, Alka Chaubey, The C.O.V.I.D19hostgenomeS.V. Consortium, Alexander Hoischen, Alan H. Beggs, Clement A. Adebamowo, Ali Andalibi, Silviu‐Alin Bacanu, Vineet Bafna, Justin Bahl, Hayk Barseghyan, Alan Beggs, Laurie Burdette, Manish Butte, Pantelis Constantoulakis, Keith A. Crandall, Siavash R. Dehkordi, Megan Dennis, Gang Fang, Olivier Fedrigo, Darren Finlay, Michael A. Goldman, Umamaheswaran Gurusamy, Vanessa Hayes, Glenn Hickey, Alexander Hoischen, Thomas Illig, Alexander Ioannidis, Erich Jarvis, Naoru Koizumi, Ravindra Kolhe, Meriem Laamarti, Celia Labranche, Sandra Leibel, Brynn Levy, Matthew Loose, Claudio Mello, Jamal Nasir, Thuy L. Phung, Chethan P. Venkatasubba Rao, Ted Ross, Nikhil S. Sahajpal, Rashmi K. Shamanna, Daniela C. Soto, Amir Trablesi, Zi‐Xuan Wang, Sion Llewelyn Williams, Victoria Wright, Hua Zhao, Michael Zody

**Affiliations:** ^1^ Division of Genetics and Genomics The Manton Center For Orphan Disease Research Harvard Medical School Boston Children's Hospital Boston Massachusetts USA; ^2^ Children's Rare Disease Collaborative Boston Children's Hospital Boston Massachusetts USA; ^3^ Department of Human Genetics Radboud University Medical Center Nijmegen the Netherlands; ^4^ CAIMed ‐ Lower Saxony Center for Artificial Intelligence and Causal Methods in Medicine (CAIMed) Hannover Germany; ^5^ Hannover Unified Biobank Hannover Medical School Hannover Germany; ^6^ Bionano Genomics San Diego California USA

**Keywords:** MIS‐C, optical genome mapping, SARS‐CoV‐2

## Abstract

Multisystem inflammatory syndrome in children (MIS‐C) is a pediatric complication of SARS‐CoV‐2 infection characterized by multiorgan inflammation and frequently by cardiovascular dysfunction. In a single‐center prospective cohort study, optical genome mapping (OGM) was performed on 14 patients, including 11 meeting CDC criteria for MIS‐C and 3 with MIS‐C–like (MIS‐CL) presentations. SVs and CNVs were filtered against population and internal OGM control databases. Seven patients (50%) harbored prioritized variants within or near genes implicated in immune regulation or SARS‐CoV‐2 response. These included intronic insertions or deletions in *ORAI1*, *STAT4*, and *ITPR1* (n = 4 patients); a heterozygous insertion disrupting *BATF*; a large deletion spanning exons 2–10 of *CFHR5*; and an upstream insertion near *DOCK2*. Application of OGM to patients with MIS‐C and MIS‐CL revealed SVs potentially impacting inflammation, COVID‐19 severity, and Kawasaki Disease susceptibility. Although causality cannot yet be assigned, the identification of rare structural variants highlights biologically plausible mechanisms that may contribute to disease heterogeneity. These findings establish the feasibility and value of OGM in the assessment of complex pediatric syndromes, such as children with MIS‐C or a severe course of SARS‐CoV‐2 infection.

## Graphical abstract

This study applies optical genome mapping (OGM) to children with multisystem inflammatory syndrome (MIS‐C), revealing rare structural and copy number variants in and near immune‐related genes, including *ORAI1*, *STAT4*, *ITPR1*, *BATF*, *CFHR5*, and *DOCK2*. Findings highlight OGM's potential as an effective method for uncovering potentially hidden genetic contributors to immune dysregulation.

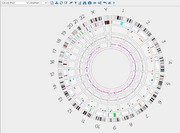

## 1 Introduction

The advent of next‐generation DNA sequencing has revolutionized our ability to interrogate patient genomes for single‐nucleotide or small insertion/deletion (indel) genetic variants that influence the development of disease. However, the contribution of large copy number variants (CNVs) and more complex structural variants (SVs), especially for multifactorial conditions, has been harder to assess. For non‐Mendelian medical conditions with presumed genetic components, association studies evaluating single nucleotide polymorphisms (SNPs) using high throughput SNP chips have occasionally been successful in identifying larger CNVs such as microdeletions and duplications at 16p11.2 in autism spectrum disorder [1], and severe early‐onset childhood obesity [2], but such methods provide limited resolution and are unable to map more complex rearrangements. Optical genome mapping (OGM) is a direct method for visualizing genomic organization at the single‐molecule level, allowing for *de novo* assembly and allele‐specific detection of balanced and unbalanced SVs [3] with high sensitivity and specificity when compared to standard‐of‐care diagnostic tests [4].

Among the many challenges that arose from the recent coronavirus disease 2019 (COVID‐19) pandemic, understanding the variable host response has been paramount. Early studies reported an association between ABO/Rh groups and the severity of COVID‐19 illness [5, 6]. However, this finding is not consistent across studies, and some do not support blood type correlation with the severity of illness [7]. A gene cluster at 3p21.31 containing several genes (including *SLC6A20, LZTFL1, CCR9, FYCO1, CXCR6*, and *XCR1* amongst others) was identified as a risk locus for respiratory failure after infection with severe acute respiratory syndrome coronavirus 2 (SARS‐CoV‐2) [8]. Other genes with variants linked to risk of developing severe or critical COVID‐19 include *TLR7* [9, 10, 11] and *FOXP4* variants [12]. An East‐Asian‐specific risk allele at the *DOCK2* region was found to confer a risk of severe COVID‐19, particularly in young individuals [13]. *ACE2*/*TMPRSS2* variability has also been discussed as a candidate for SARS‐CoV‐2 susceptibility [14]. Higher levels of *ACE2* expression were associated with potentially better outcomes of SARS‐CoV‐2 for both sexes [15], and severe cases had significantly higher *ACE1* DD genotype and D allele frequencies [16]. However, another study found no statistical difference in *ACE2* variants between control and severe cases [17]. Studies of hospitalized adults with severe COVID‐19 have identified deleterious genetic variants impairing type I interferon signaling in up to 3.5% of patients [18, 19], and additional type I interferon (IFN‐I)‐associated genes have been implicated through GWAS studies [20].

**FIGURE 1:**
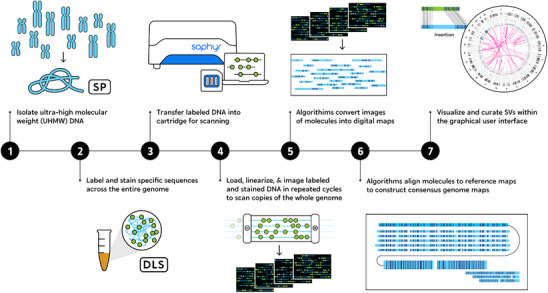
Schematic overview of the optical genome mapping (OGM) process. Steps include: (1) extraction of ultra‐high molecular weight DNA using Bionano Prep SP, (2) fluorescent labeling with Direct Labeling Enzyme 1 (DLE‐1), (3) loading of labeled DNA onto a Saphyr cartridge, (4) imaging of DNA molecules in nanochannels, (5) processing of images to extract DNA molecule maps and fluorophore positions, (6) assembly into consensus maps via *de novo* and rare variant pipelines, and (7) variant visualization using Bionano Access software v1.6.1. Data are presented as a schematic workflow created by the authors; no statistical tests were performed (*n = 14 patients; 11 MIS‐C, 3 MIS‐CL*).

**FIGURE 2:**
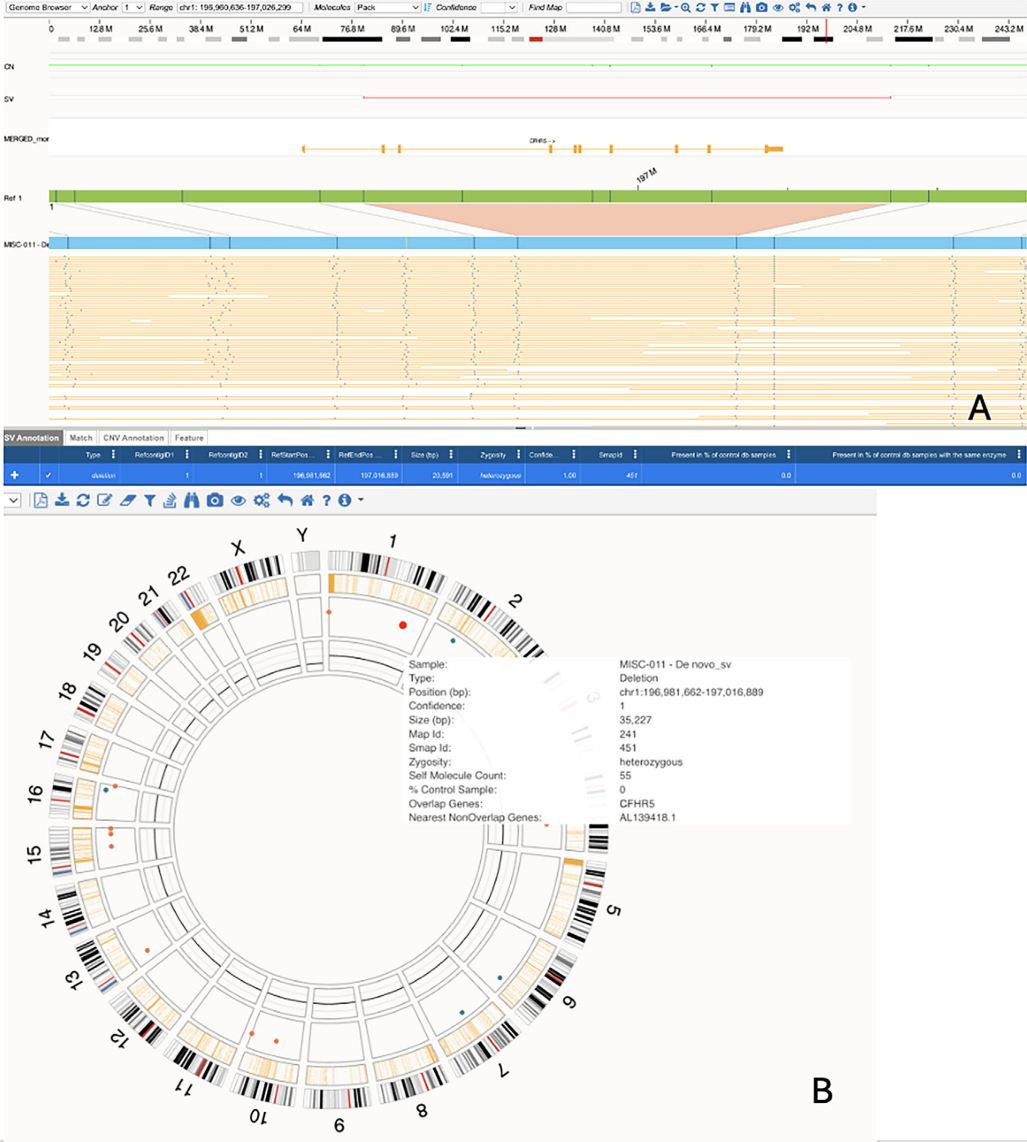
Copy number variant in *CFHR5* detected by optical genome mapping (OGM). (A) Genome browser view of the *CFHR5* locus in patient MISC011 showing a ∼20.6 kb heterozygous deletion spanning exons 2–10. (B) Circos plot summarizing the distribution of rare structural variants across the genome for this patient. Data are representative of OGM readouts; no statistical tests were performed (*n = 1 patient*).

Multisystem inflammatory syndrome of children (MIS‐C) is a rare life‐threatening post‐infection complication of SARS‐CoV‐2 exposure occurring in individuals younger than 21 years of age [21, 22]. As defined by the Centers for Disease Control and Prevention, the diagnostic criteria include fever, elevated inflammatory marker levels, multisystem organ involvement, and SARS‐CoV‐2 infection or exposure within 4 weeks of symptoms without an alternative diagnosis [23]. Symptoms commonly include fever, rash, gastrointestinal symptoms, coagulopathy, cardiac dysfunction, and/or shock [21, 22]. Nearly all patients with MIS‐C have detectable antibodies to SARS‐CoV‐2, and many have detectable SARS‐CoV‐2 virus by reverse transcriptase polymerase chain reaction (RT‐PCR) testing [21, 22]. The clinical features of MIS‐C overlap with those of acute COVID‐19 and those of the pediatric Kawasaki disease (KD) [21, 22]. Clinical features that overlap incompletely with MIS‐C can be considered MIS‐C “Like”, abbreviated “MIS‐CL”. MIS‐C is not associated with preexisting cardiopulmonary, autoimmune, and/or immune, or hematologic disease, and its genetic basis is largely unknown [21]; however, deficiencies of OAS1, OAS2, or RNase L have been described in approximately 1% of MIS‐C patients [24]. Other studies employing next‐generation sequencing in MIS‐C cohorts have implicated single‐nucleotide variants (SNVs) in the *SOCS1*, *XIAP*, and *CYBB* genes and variation in IFN‐I genes [26] as predisposing factors to the development of MIS‐C (Figures 1 and 2).

To complement these studies and evaluate the influence of large CNVs and more complex SVs on disease course, we previously applied OGM technology to a cohort of 52 patients with severe COVID‐19 [27]. We identified seven SVs involving genes implicated in two key host‐viral interaction pathways: innate immunity and inflammatory response, and viral replication and spread in nine patients (17%), of which SVs involving *STK26* and *DPP4* genes were the most intriguing candidates. Here, we employed OGM (using the Saphyr System by Bionano Genomics, Inc., San Diego, CA) to examine the role of SVs in a cohort of patients with MIS‐C.

## 2 Methods

### 2.1 Patient Population

A total of 11 children and adolescents with suspected or diagnosed MIS‐C using CDC criteria [28] and three with MIS‐CL presentations at Boston Children's Hospital were enrolled between 15 March 2020 and 31 December 2021. Patients or their guardians all provided informed consent under an approved protocol of the Boston Children's Hospital Institutional Review Board. Inclusion criteria were: 1) age< 21 years and 2) positive SARS‐CoV‐2 test during illness RT‐PCR or antibody), 3) suspected MIS‐C or MIS‐CL, and 4) without presumed causative findings on short read next generation sequencing (NGS).

### 2.2 Isolation of Ultra‐High Molecular Weight DNA, Labeling and Analysis

Peripheral blood from critically ill patients was drawn and used to isolate ultra‐high molecular weight (UHMW) DNA prepared for analysis on the Bionano Genomics Saphyr system (Bionano Genomics, San Diego, USA, #90001) (Figure 1) following the manufacturer's protocols (Bionano Genomics, San Diego, USA, #90057). Briefly, a frozen whole blood aliquot (650 µL) was thawed, and cells were counted using HemoCue (Microcuvettes and WBC analyzer, HemoCue Holding AB, Ängelholm, Sweden). Subsequently, a blood aliquot comprising approximately 1.5 million nucleated white blood cells was centrifuged and digested with Proteinase K. DNA was precipitated using isopropanol and washed using buffers (buffer A and B), while the DNA remained adhered to the nanobind magnetic disk. The UHMW‐bound DNA was resuspended in elution buffer and quantified with Qubit dsDNA BR assay kits (ThermoFisher Scientific, San Francisco, USA, #Q32850) using a Qubit fluorimeter (ThermoFisher Scientific, San Francisco, USA, #Q33226).

DNA labeling was performed following the manufacturer's protocols (Bionano Genomics, USA, #80005) in which Direct Labeling Enzyme 1 (DLE‐1) fluorescently labels a specific 6‐base sequence motif (CTTAAG) in the genome using 750 ng of purified high molecular weight DNA. Labeled DNA was quantified using Qubit dsDNA HS assay kits (ThermoFisher Scientific, San Francisco, USA, #Q32851) and loaded onto flow cells of Saphyr chips (Bionano Genomics, USA, #20366) for imaging. The fluorescently labeled DNA molecules were imaged on the Saphyr instrument after the labeled DNA molecules were electrophoretically linearized in the nanochannel arrays. Effective (mappable) genome coverage was assessed for the tested samples after evaluating the molecule quality metrics. The quality control metrics for each sample were assessed against the recommended molecule map rates of greater than 70% and molecule N50 values greater than 230 kb in size.

### 2.3 Optical Genome Mapping Workflow and Bioinformatics Pipelines

The bioinformatics architecture for OGM includes two pipelines (*de novo* assembly and rare variant analysis). The *de novo* pipeline assembles single molecules using the alignment of sequence labels in pairwise alignments. The aligned molecules assemble a *de novo* consensus map, which is then compared with the in silico DLE1 hg38 reference map. The *de novo* pipeline is able to identify SVs greater than 500 bp. In contrast to the *de novo* pipeline, the rare variant pipeline aligns single molecules against the hg38 reference map. Any molecules with SVs are then clustered, and consensus maps are realigned against the hg38 reference. This pipeline detects SVs greater than 5000 bp at a molecule variant allele frequency (VAF) greater than 5%.

Deletions, insertions, duplications, and inversions, in addition to inter‐ and intra‐chromosomal aberrations, were annotated by the *de novo* and rare variant pipelines using the Bionano Access software version 1.6.1.

Filtering for both pipelines included SVs with VAF >5% and a minimum of ten self‐molecules. CNVs and SVs were queried in gnomAD (v4.1.0 (N = 63,046 genomes, N = 464,297 exomes)) to determine if overlapping CNVs or SVs were identified using NGS. CNVs and SVs identified in greater than 1% of the database were not considered further. To filter out common variation that is only detected using OGM technology and would not be in NGS‐based allele frequency databases due to technology‐specific platform limitations, the results were compared to the Bionano Technologies internal database. This cohort of OGM‐run control human genomes has no reported disease phenotypes (N = 285). Variants of interest were also compared against our internal OGM database (N = 700). SVs occurring in more than 1% of either database were not investigated further.

Finally, the pipeline outputs were manually assessed for pathogenicity using ClinVar, PubMed, and internal databases. Genes involved in SVs and CNVs were assessed using PubMed and clinical databases for association with phenotypes and diseases, in particular KD and SARS‐CoV‐2, though all associations were reviewed. Genetic constraint and haploinsufficiency scores for each gene were examined using gnomAD and Decipher (accessed January 1, 2025).

### 2.4 Statistical Analysis

Given the small sample size of this exploratory cohort, no formal hypothesis testing was performed, and all results are descriptive and hypothesis‐generating. Quality control metrics and SV counts were summarized in Bionano Access (v1.6.1) and R (v4.2.2) using descriptive statistics (mean, median, and range). Data are presented as mean ± standard deviation (SD) unless otherwise indicated. The sample size (n) for each experiment or dataset is reported in the corresponding figure legend. No data transformation, normalization, or outlier exclusion was applied, and no inferential statistical tests were conducted.

## 3 Results

### 3.1 Demographics

Fourteen children and adolescents were enrolled, including 11 who met full CDC criteria for MIS‐C and 3 with MIS‐CL presentations (Table 1). The cohort included 9 males and 5 females, spanning ages from infancy through 20 years. Ethnic and racial distribution was predominantly non‐Hispanic White (n = 6), with additional representation from Asian (n = 1) and other/unknown backgrounds. Comorbidities were uncommon, with 9/14 patients (64%) previously healthy at baseline. Severe disease manifestations were frequent: 5/14 patients (36%) developed shock requiring vasopressors, and 6/14 (43%) exhibited decreased cardiac function (Table 2).

**TABLE 1:** Demographics and clinical characteristics of the study population (*n = 14 patients; 11 MIS‐C, 3 MIS‐CL*). Clinical variables include ethnicity, age group, prior health status, and severe disease features such as shock requiring vasopressors and decreased cardiac function. Data are descriptive; no inferential statistical testing was performed.

ID	Diagnosis^1^	Ethnicity^2^	Race	Gender	Age group (years)	Previously healthy	Shock requiring vasopressors	Decreased Cardiac Function
MISC001	MIS‐C	N	Other	Male	6–11	No	Yes	Yes
MISC015	MIS‐C	N	Asian	Male	12–20	Yes	No	No
MISC011	MIS‐C	N	White	Male	6–11	No	No	Yes
MISC004	MIS‐C	H	White	Female	6–11	No	No	No
MISC013	MIS‐C	N	White	Male	6–11	Yes	No	No
MISC034	MIS‐C	N	White	Female	12–20	Yes	Yes	Yes
MISC002	MIS‐C	N	Other	Male	0–5	Yes	No	Yes
MISC009	MIS‐C	N	White	Male	0–5	Yes	No	No
MISC018	MIS‐C	N	Other	Male	6–11	Yes	Yes	Yes
MISC021	MIS‐C	N	White	Female	0–5	Yes	Yes	Yes
MISC041	MIS‐C	Unknown	Unknown	Male	6–11	No	No	No
MISC008	MIS‐C L^3^	H	Other	Female	6–11	No	No	No
MISC017	MIS‐C L^4^	Unknown	Unknown	Male	12–20	No	No	Yes
MISC030	MIS‐C L^5^	N	Other	Female	0–5	Yes	No	No

^1^ MIS‐C = meets CDC criteria for MIS‐C. MIS‐C‐L = MIS‐C‐Like.

^2^ N = Not Hispanic or Latino, H = Hispanic or Latino.

^3^ MIS‐C‐like, Post‐viral syndrome antibody‐positive.

^4^ Suspected MIS‐C but antibody negative—Mother had COVID one month prior he was negative.

^5^ Per Rheumatology, the greatest clinical concern was for post‐COVID inflammatory process on the spectrum of but not meeting criteria for MIS‐C.

**TABLE 2:** Genes involved in rare copy number or structural variants identified in the cohort with known or putative roles in immune regulation or COVID‐19 severity (n = 14 patients). Variants are annotated by genomic coordinates (hg38), size, pLI score, predicted haploinsufficiency probability, and frequency in internal and public databases. References correspond to prior reports linking each gene to COVID‐19 or immune phenotypes. Data are descriptive; no statistical tests were performed.

Gene	ID	Structural variant or copy number description	Size (bp)	SV Coordinates (hg38)	pLI of gene^*^	pHaplo: Predicted Probability of Haploinsufficiency [61, 62]	Bionano Database frequency	Reference for gene link to COVID‐19 severity involvement
*ORAI1*	MISC030	Heterozygous intronic insertion	508	NC_000012.12:g.(121630784_ 121630785)ins508	0	0.51	0.39%	[35]
*STAT4*	MISC001	Heterozygous intronic insertion	5936	NC_000002.12:g.(191078656_ 191096894)ins5936	0.93	0.52	0.63%	[43]
*ITPR1*	MISC002	Heterozygous intronic deletion	1114	NC_000003.12:g.(4634056_ 4637180)del1114	1	1.00	0.53%	[63]
	MISC004	Heterozygous intronic deletion within intron 4	706	NC_000003.12:g.(4585302_ 4588106)del706			0.49%	
*BATF*	MISC041	Heterozygous insertion involving exon 3	2,363	NC_000014.12:g.(75539993_ 75569126)ins2363	0.56	0.76	0.51%	[64]
*CFHR5*	MISC011	Heterozygous deletion of exons 2‐10	20,591	NC_000001.12:g.(196981662_ 197016889)del20591	0	0.03	0	[56, 57]
*DOCK2*	MISC015	Insertion located 5,218 bp 5’ of *DOCK2*	26,293	NC_000005.10:g.(169,624,051‐169,632,057)ins26293	1	0.99	0	[13]

* pLI from gnomAD, checked September 1, 2025.

### 3.2 Optical Genome Mapping Performance

High‐quality U DNA was successfully isolated from all samples, with mean effective coverage of ∼400X and molecule N50 values exceeding manufacturer thresholds (Tables S1a–c). Average mapping rate across samples was 87.7%, and quality control metrics indicated robust data suitable for downstream structural variant detection.

### 3.3 Structural Variant Burden

After filtering against gnomAD v4.1.0 and two OGM control databases, each patient harbored, on average 48 rare structural variants, with a median of 47 (range: 41–56). These included a mean of 20 deletions, 25 insertions, and 3 other rearrangements per individual. Prioritization based on gene function and prior literature revealed that six patients, representing 43% of the cohort, carried rare SVs or CNVs overlapping genes implicated in immune regulation or SARS‐CoV‐2 response. One patient (7% of the cohort) carried an insertion not encompassing any genes, but located 5’ of a gene implicated in SARS‐CoV‐2 response (Table 2).

In patient MISC030, OGM identified a heterozygous intronic insertion within the *Calcium release‐activated calcium modulator 1* (*ORAI1*) gene (chr12:121630785–121632856, 508 bp). The patient was a young female (0–5 years), previously healthy, and did not experience shock or cardiac dysfunction.

In patient MISC001, we detected a heterozygous intronic insertion of unknown origin in the *Signal transducer and activator of transcription 4* (*STAT4*) gene (chr2:191078656‐191096894, 5936 bp). This patient (male, 6–11 years) experienced severe disease, with both shock requiring vasopressors and decreased cardiac function.

Two patients carried non‐overlapping intronic deletions in the *Inositol 1,4,5‐triphosphosphate receptor, type 1* (*ITPR1*) gene. Patient MISC002 harbored a heterozygous intronic deletion of 1114 bp in intron 2 (chr3:4634056‐4637180). The patient, a previously healthy young male (0–5 years), had decreased cardiac function but no shock. Patient MISC004 had a heterozygous intronic deletion of 706 bp in intron 4 (chr3:4585302‐4588106). This female patient (6–11 years) had neither shock nor cardiac involvement.

Patient MISC041 harbored a heterozygous insertion within the *Basic leucine zipper transcription factor, ATF‐like* (*BATF*) gene (chr14:75539993‐75569126, 2,363 bp). BATF encodes a transcription factor belonging to the AP‐1 family. The identified insertion disrupts exon 3. This patient (male, 6–11 years) had a disease course without shock or decreased cardiac function.

In patient MISC011, a large heterozygous deletion of 20591 bp was identified in the *Complement factor H‐related 5* (*CFHR5*) gene (chr1:196981662‐197016889). This deletion spans exons 2–10 of the gene (Figure 2). The patient (male, 6–11 years) exhibited decreased cardiac function but did not develop shock.

Patient MISC015 was identified as having a heterozygous 26293 bp insertion located approximately 5218 bp 5’ of the *Dedicator of cytokinesis 2* (*DOCK2)* gene (chr5:169,624,051‐169,632,057). The insertion region includes 5 cis‐Regulatory Elements (cCREs) with distal enhancer‐like signatures (ENCODE Accessions: EH38E2430099, EH38E2430100, EH38E2430101, EH38E2430102, and EH38E2430105) [29]. This patient (male, 12–20 years, Asian ancestry) was previously healthy and did not develop shock or cardiac dysfunction.

## 4 Discussion

While we and others have previously used OGM to identify variants in genes representing potentially novel mechanisms for severe SARS‐CoV‐2 [25, 27], assessment of potential disease‐modifying SVs and CNVs in the genomes of patients with MIS‐C has not been extensively reported. In this study, we applied OGM to a cohort of children with MIS‐C and MIS‐C‐like syndromes and identified SVs and CNVs in several genes implicated in immune regulation, inflammatory signaling, and host responses to viral infection. The potential role(s) of these variants in the etiology of MIS‐C or other aspects of these patients’ response to SARS‐COV‐2 infection remain unclear and will require extensive functional follow‐up. However, their identification through OGM illustrates the ability of this technology to discover previously undetectable or underrepresented forms of genetic variation.

### 4.1 ORAI1

The intronic insertion in *ORAI1* is of interest due to the role of ORAI1 in KD; there is a significant association between an *ORAI1* SNP (rs141919534) and development of the disorder [30, 31]. Also notable is ORAI1's central role in store‐operated calcium entry (SOCE) and T cell signaling [32, 33]. Disrupted calcium signaling has previously been implicated in both immunodeficiency syndromes and susceptibility to viral infection, including SARS‐CoV‐2 [32, 34, 35].

Biallelic sequence variants in *ORAI1 *are also associated with infections in severe combined immunodeficiency due to impaired T cell activation [36], and a case of ORAI1 deficiency presented with immunodeficiency and residual T cell function [37]. Orai1 deletion in mice causes increased susceptibility to SARS‐COV‐2 infection through impairment of tonic IFN‐I signaling, indicating a potential role played by ORAI1 in resistance to SARS‐COV‐2 [38]. Human cells lacking ORAI1 also exhibited reduced IFN‐I signaling and increased susceptibility to SARS‐CoV‐2 infection [39]. This suggests that it may be specific to rare *ORAI1* loss‐of‐function variants influencing the variability in COVID‐19 symptoms among patients, though the role of host cell calcium signaling during viral infections remains poorly understood overall [35]. Other studies found no significant link between *ORAI1* genetic variants and susceptibility to SARS‐CoV‐2 infection or COVID‐19 severity [32, 39]. Although the functional impact of the intronic insertion in the MIS‐C patient remains unclear, this finding raises the possibility that altered calcium flux contributes to aberrant immune activation in MIS‐C.

### 4.2 STAT4

While unknown, the *STAT4* intronic insertion could possibly result in dysregulation of cytokine signaling pathways. *STAT4* encodes a protein belonging to the family of signal transducers and activators of transcription (STAT) proteins, which are critical transcription factors involved in numerous biological processes [40, 41]. STAT4 plays a pivotal role in promoting antiviral IFN‐I and interferon‐gamma (type 2) production, as demonstrated in challenge models with single‐stranded RNA (ssRNA) and DNA viruses [40, 41]. It transduces signals mediated by interleukin‐12 (IL‐12), interleukin‐23 (IL‐23), thereby contributing to T helper 1 (Th1) polarization.

The importance of STAT4 in cytokine regulation is underscored by its association with several autoimmune diseases. Conditions such as systemic lupus erythematosus (SLE), rheumatoid arthritis (RA), inflammatory bowel disease (IBD), and Sjögren's syndrome have been linked to SNPs in the *STAT4* gene [42].

In the context of SARS‐CoV‐2, *STAT4* has been identified as a target of SARS‐CoV‐2 microRNAs (miRNAs) [43] and has been documented to be upregulated in patients with the disease [44]. STAT4 is also integral to the JAK/STAT signaling pathway, which SARS‐CoV‐2 is known to dysregulate. This dysregulation contributes to the hyperinflammatory state observed in severe COVID‐19 cases, often referred to as a “cytokine storm.” Importantly, the cytokine storm is thought to be important in MIS‐C pathogenesis and disease progression [45]. Therefore, variation in *STAT4* may act as a modifier of hyperinflammatory responses in this context.

### 4.3 ITPR1

Two patients harbored intronic deletions in *ITPR1*, a gene encoding a receptor involved in intracellular calcium release from the endoplasmic reticulum [46]. While the deletions are non‐coding, disruption of splicing or regulatory domains is possible. ITPR1 is expressed in a wide range of tissue types and shows a higher level of expression within the hippocampus, caudate, putamen, and cerebellar Purkinje cells. ITPR1 is composed of an N‐terminal IP3 binding domain, a central regulatory domain, and a C‐terminal transmembrane pore [47]. A polymorphism in *ITPKC*, encoding inositol‐triphosphate three kinase C that is part of the same calcium signaling pathway, has been described in KD predisposition [48].

It is interesting that SVs impacting two genes that regulate calcium homeostasis were identified in this small cohort (*ITPR1* and *ORAI1*). Altered calcium signaling may lead to improper activation of autoreactive immune cells or exacerbate inflammatory responses. Together with the *ORAI1* finding, these results suggest that abnormalities in calcium signaling may represent a convergent mechanism predisposing to exaggerated immune responses following SARS‐CoV‐2 exposure.

### 4.4 BATF

The BATF protein belongs to the AP‐1 family of transcription factors and is expressed in T cells, B cells, and dendritic cells. In T cells, BATF is activated upon TCR stimulation and plays an essential role in T cell differentiation, particularly the development of functional Th17 T cells, and operates as a differentiation checkpoint in early effector CD8⁺ T cells [49]. BATF contributes both to effector function and to exhaustion of T cells depending on the immunological context, and also regulates antibody class‐switching through its requirement in follicular helper T cells and B cells [50].

In this study, patient MISC041 carried a heterozygous insertion involving exon 3 of *BATF*, predicted to alter transcript structure or expression, although the precise molecular consequence remains unknown. Depending on how the insertion affects transcription or splicing, it could theoretically lead to *BATF* haploinsufficiency or, alternatively, increased or dysregulated *BATF* expression if the inserted sequence introduces enhancer elements or stabilizes transcription. Without functional assays, both possibilities remain plausible.

*BATF* expression is upregulated in severe COVID‐19 [51], and both insufficient and excessive BATF activity could plausibly contribute to immune imbalance. Reduced expression could impair the ability of exhausted CD8⁺ T cells to re‐enter effector states during prolonged antigenic exposure, while overexpression might exacerbate inflammatory signaling through the AP‐1 network, promoting cytokine overproduction. Either mechanism could sustain the aberrant immune activation characteristic of MIS‐C [52, 53, 54].

Although no prior reports directly link germline *BATF* variation to SARS‐CoV‐2 outcomes, these findings suggest that perturbation of BATF‐regulated transcriptional programs may alter the balance between effective antiviral immunity and pathological inflammation in MIS‐C. Functional studies will be essential to define whether this insertion causes haploinsufficiency, overexpression, or a more complex regulatory disruption.

### 4.5 CFHR5

CFHR5 competes with CFH (Factor H) for binding to the immune system inhibitor CHFR3b. It is thought that when CFHR5 blocks CFH, it disrupts the complement system balance and allows for excessive activation, leading to inflammation [55]. Recent studies have highlighted the association between elevated plasma levels of CFHR5 and increased severity of COVID‐19 [56, 57]. In addition, it was shown that auto‐antibodies against factor H are elevated in MIS‐C patients, leading to complement dysregulation [58].

A deletion in *CFHR5* is a seemingly paradoxical observation, and the impact of this deletion, if any, is unclear. It is possible that a large deletion could disrupt the regulatory roles of CFHR5 rather than simply reducing its immune‐activating function through a decrease in competitive binding ability. It is possible that the expression of an altered or truncated form of the protein results in abnormal interactions within the complement pathway. Alternatively, loss or dysfunction of CFHR5 might allow other complement regulators or activators to increase their activity. It is possible that partial gene deletion, which intuitively might be expected to decrease immune activation, could instead lead to production of a dominantly acting truncated protein that disrupts the complement regulatory network and leads to immune system dysregulation and heightened inflammatory responses. It is also possible that the deletion also deletes regulatory elements that influence the expression of other genes within the locus (e.g., factor H‐related complement factors).

### 4.6 DOCK2

Finally, the insertion 5’ of the *DOCK2* gene is of interest, as the insertion region contains several transcriptional control elements that may influence *DOCK2* expression. The carrier of this insertion is of Asian descent, and previous investigations into *DOCK2* have highlighted an East Asian specific risk allele for severe COVID‐19, particularly affecting individuals below 65 years of age [13]. This observation bears further investigation as an insertion that disrupts any of these distal enhancers for *DOCK2* could significantly alter its expression, particularly in immune cell lineages, leading to impaired interferon signaling and abnormal immune responses. The magnitude of effect would depend on whether the enhancers act redundantly or synergistically, and whether chromatin architecture is altered.

Taken together, these findings support a model in which diverse genomic alterations may converge on immune pathways that regulate calcium signaling, cytokine production, T cell differentiation, and complement activity. Each of these processes has been implicated in the hyperinflammation and multi‐organ involvement characteristic of MIS‐C. While causality cannot be inferred from the present cohort, the results emphasize the value of OGM for revealing structural variation in patients with unexplained inflammatory syndromes. Larger cohorts, parental studies to determine inheritance, and functional assays will be required to clarify the pathogenic role of these SVs.

### 4.7 Limitations

A limitation of this study is the relatively small sample size of the cohort and the limited number of OGM controls for comparison, which requires that we interpret the findings with caution. Second, due to the nature of how our cohort was ascertained and consented, we were not able to recontact the individuals to obtain additional biological samples, phenotypic details, or family history. Thus, we are unable to functionally assess the impact or even determine if the CNVs and SVs detected are *de novo* or inherited, which would assist our assessment of their pathogenicity. Another limitation is the completeness of available phenotypic data. Available clinical data were focused exclusively on their clinical course and response to SARS‐CoV‐2 infection and may not have included other subtle findings potentially referable to the SVs and impacted genes.

We also identified apparently rare variations, though the increasing utilization of OGM and development of larger population‐wide SV databases may reveal that some of this variation is not rare in the general population. Misclassifications due to a lack of NGS data have occurred for several variants in the *SCN1A* gene [59] and variants in several cardiac genes [60], and this is a risk during the early stages of adoption of any new technology. Increasing the sizes and diversity of genomic datasets for novel technologies such as OGM is a high priority to allow proper classification of variants based on their population frequencies.

## 5 Conclusions

This systematic application of OGM to MIS‐C and MIS‐CL showed that half of the cohort carried rare variants in genes implicated in immune regulation or SARS‐CoV‐2 responses, including alterations in calcium signaling, cytokine regulation, and complement pathways. While causality cannot yet be assigned, these findings highlight biologically plausible mechanisms underlying MIS‐C heterogeneity and support the diagnostic value of OGM in complex pediatric syndromes, such as children with MIS‐C or severe SARS‐CoV‐2 infection.

## Funding

The Eunice Kennedy Shriver National Institute of Child Health and Human Development grant P50‐HD105351.

## Conflicts of Interest

A.P., J.M.R., A.H., and A.C. are or were salaried employees and hold equity in Bionano Genomics, Inc. The remaining authors declare no conflicts of relevance to this study.

## Supporting information




**Supporting File**: ggn270017‐sup‐0001‐SuppMat.docx.

## Data Availability

The data that support the findings of this study are available from the corresponding author upon reasonable request. Optical genome mapping data are not publicly available due to consent restrictions.
