# Supplementary material for: Rare Structural Variants Uncovered by Optical Genome Mapping in Multisystem Inflammatory Syndrome in Children (MIS‐C)
Source: Adv Genet (Hoboken). 2025 Dec 8;6(4):e00023. doi: 10.1002/ggn2.202500023 (PMC12747551; doi:10.1002/ggn2.202500023)
Supplement: Supplementary file 1 — Supporting File: ggn270017‐sup‐0001‐SuppMat.docx. [file GGN2-6-e00023-s001.docx]

### **Supplemental Table S1a.** Key optical genome mapping parameters for the MIS-C cohort. Metrics include enzyme recognition site, total reference length, and mapping parameters for all 14 samples. Data reflect technical specifications of the OGM platform; no statistical tests were performed (n = 14 samples).

| **Metric** | **units** |  |
| --- | --- | --- |
| Name of the reference genome this sample was aligned to. | Reference | hg38_DLE1_0kb_0labels.cmap |
| Total length of reference sequence | Reference Length | 3088269832 |
| Name of the enzyme used in this sample. | Enzyme | DLE-1 |
| Recognition sequence of the enzyme used. | Site | CTTAAG |
| Label color used for detection. | Label color | BNGFLGR001 |

**Supplemental Table S1b. OGM Summary Statistics**

Summary statistics for optical genome mapping performance across the MIS-C cohort (*n = 14 patients*). Values represent averages and standard deviations for N50 molecule lengths, total DNA yield, map rate, and effective coverage. Data are presented as mean ± SD. No inferential testing was performed.

| **Metric** | **units** | **Average** | **Std Dev** |
| --- | --- | --- | --- |
| N50 of the molecules that are 20kbp or longer) | N50 (>= 20 kbp) | 205.66 | 54.73 |
| Total amount of DNA from molecules that are 20 kbp or longer | Total DNA (>= 20kbp) | 1659.49 | 719.45 |
| N50 of DNA molecules that are 150kbp or longer | N50 (>= 150kbp) | 266.54 | 33.67 |
| Total amount of DNA from molecules that are 150kbp or longer | Total DNA (>= 150kbp) | 739.43 | 327.12 |
| Same as other N50 fields, but molecules must have at least 9 labels | N50 (>= 150kbp and min sites >=9) | 268.18 | 34.15 |
| Same as other Total DNA fields, but molecules must have at least 9 labels | Total DNA (>= 150kbp and min sites >= 9) | 711.09 | 313.81 |
| Percentage of molecules that are 150kbp or longer mapped to the reference | Map rate | 87.69% | 0.06 |
| Total amount of aligned DNA divided by the size of the reference genome times the map rate. | Effective coverage | 202.05 | 91.36 |
| Average number of labels per 100 kbp for the molecules that are 150kbp or longer | Average label density (>= 150kbp) | 15.23 | 0.42 |
| Constant term in sizing error relative to reference | Site SD | 0.11 | 0.01 |
| Quadratic term in sizing error relative to reference | Relative SD | 0.012 | 0.00 |
| Calculated base pairs per pixel in the alignment by comparing molecules to the reference. | Base pairs per pixel | 478.42 | 10.35 |
| Percentage of reference labels absent in molecules | Negative label variance (NLV) | 8.08 | 1.79 |
| Percentage of labels absent in reference | Positive label variance (PLV) | 3.39 | 0.82 |
| Linear term in sizing error relative to reference | Scaling SD | 0.01 | 0.01 |
| integrity_num |  | 0.10 | 0.03 |

**Supplemental Table S1c**. **Molecule Quality Report (MQR)** Optical genome mapping molecule quality metrics per individual sample (*n = 14 patients*). Each column corresponds to one patient sample. Reported values include N50, total DNA yield, mapping rate, label density, and alignment error metrics. Data are descriptive and quality-control based; no statistical tests were performed.

| Metric | units | misc001 | misc009 | MISC008 | misc011 | misc012 | misc13 | misc15 | misc017 | misc018 | misc021 | misc030 | misc034 | misc002 | misc004 | misc041 |
| --- | --- | --- | --- | --- | --- | --- | --- | --- | --- | --- | --- | --- | --- | --- | --- | --- |
|  |  | COVP-001-026-841 | CovP-001-067-244 | cov-0365-316 | COVP-001-045-465 | COVP-001-066-606 | COVP-001-063-897 | COVP-001-039-355 | COVP-001-027-354 | COVP-001-072-638 | COVP-001-074-411 | COVP-001-073-014 | COVP-001-064-772 | COVP-001-065-438 | COVP-001-060-604 | COVP-001-078-803 |
| N50 of the molecules that are 20kbp or longer) | N50 (>= 20 kbp) | 241.13 | 221.09 | 269.25 | 267.38 | 162.38 | 202.28 | 233.51 | 136.13 | 134.45 | 146.25 | 257.57 | 203.63 | 189.75 | 258.07 | 175.5 |
| Total amount of DNA from molecules that are 20 kbp or longer | Total DNA (>= 20kbp) | 1,932.99 | 2,099.67 | 749.59 | 797.12 Gbp | 1,189.05 Gbp | 907.74 Gbp | 858.4 Gbp | 1,373.76 Gbp | 1,402.65 Gbp | 1,276 Gbp | 897.33 Gbp | 916.29 Gbp | 601.66 Gbp | 524.08 Gbp | 1,029.56 Gbp |
| N50 of DNA molecules that are 150kbp or longer | N50 (>= 150kbp) | 279.38 | 268.88 | 311.25 | 311.63 | 247.13 | 259.22 | 295.11 | 232.76 | 238.13 | 246.36 | 316.5 | 262.86 | 231.75 | 286.5 | 237.38 |
| Total amount of DNA from molecules that are 150kbp or longer | Total DNA (>= 150kbp) | 1,503.66 | 1,516.99 | 600.27 | 634.06 | 646.2 | 611.95 | 617.16 | 618.21 | 624.53 | 622.34 | 676.5 | 611.68 | 403.42 | 438.19 | 611.63 |
| Same as other N50 fields, but molecules must have at least 9 labels | N50 (>= 150kbp and min sites >=9) | 281.25 | 270.38 | 313.5 | 313.98 | 249 | 260.63 | 297.38 | 234 | 239.25 | 247.5 | 318.75 | 264.08 | 232.88 | 288.02 | 238.53 |
| Same as other Total DNA fields, but molecules must have at least 9 labels | Total DNA (>= 150kbp and min sites >= 9) | 1,442.28 | 1,458.90 | 575.72 | 607.27 | 626.18 | 583.21 | 591.43 | 597.82 | 602.97 | 607.12 | 654.19 | 589.5 | 389.19 | 420.74 | 578.42 |
| Percentage of molecules that are 150kbp or longer mapped to the reference | Map rate | 88.20% | 85.70% | 92.30% | 93.50% | 84% | 92.50% | 92.20% | 83.90% | 83.10% | 84.20% | 91.40% | 89.10% | 86.70% | 94.30% | 87.80% |
| Total amount of aligned DNA divided by the size of the reference genome times the map rate. | Effective coverage | 411.91 | 404.85 | 172.07 | 183.86 | 170.32 | 174.68 | 176.57 | 162.41 | 162.25 | 165.53 | 193.61 | 170.08 | 109.26 | 128.47 | 164.45 |
| Average number of labels per 100 kbp for the molecules that are 150kbp or longer | Average label density (>= 150kbp) | 15.23 | 15.34 | 14.97 | 15.01 | 15.4 | 14.52 | 14.98 | 15.51 | 15.34 | 15.64 | 15.34 | 14.94 | 15.9 | 15.39 | 15.05 |
| Constant term in sizing error relative to reference | Site SD | 0.12 | 0.11 | 0.11 | 0.1 | 0.11 | 0.12 | 0.11 | 0.097 | 0.11 | 0.12 | 0.1 | 0.11 | 0.1 | 0.11 | 0.13 |
| Quadratic term in sizing error relative to reference | Relative SD | 0.016 | 0.012 | 0.013 | 0.014 | 0.012 | 0.01 | 0.011 | 0.01 | 0.011 | 0.012 | 0.013 | 0.012 | 0.013 | 0.011 | 0.027 |
| Calculated base pairs per pixel in the alignment by comparing molecules to the reference. | Base pairs per pixel | 486 | 467.76 | 477.41 | 481.85 | 477.01 | 478.04 | 482.08 | 473.97 | 465.16 | 473.82 | 476.31 | 460.15 | 484.23 | 483.96 | 508.77 |
| Percentage of reference labels absent in molecules | Negative label variance (NLV) | 8.95 | 8.04 | 7.52 | 7.54 | 8.48 | 9.72 | 8.15 | 6.49 | 6.87 | 7.35 | 7.15 | 6.87 | 6.96 | 6.89 | 10.52 |
| Percentage of labels absent in reference | Positive label variance (PLV) | 3.11 | 3.44 | 3.04 | 2.3 | 3.22 | 2.45 | 2.63 | 4.32 | 4.7 | 4.49 | 3.72 | 3.59 | 4.57 | 3.45 | 3.02 |
| Linear term in sizing error relative to reference | Scaling SD | 0.02 | 0.014 | 0 | 0 | 0 | 0 | 0.006 | 0.0023 | 0.0084 | 0 | 0 | 0 | 0.0093 | 0.0015 | 0.048 |
